# Specific carbohydrate diet versus Mediterranean diet in adult patients with mild to moderate ulcerative colitis: a randomized controlled-feeding trial

**DOI:** 10.3389/fnut.2026.1838160

**Published:** 2026-07-10

**Authors:** Albert Sheng-Yin Chen, Long H. Nguyen, Brianna Gray, Katherine Williams, Jenny Gurung, Lauren Canha, Jessica McGoldrick, Jane Hubbard, Hamed Khalili

**Affiliations:** 1 Clinical and Translational Epidemiology Unit, Massachusetts General Hospital and Harvard Medical School, Boston, MA, United States; 2 Division of Gastroenterology, Massachusetts General Hospital, Harvard Medical School, Boston, MA, United States; 3 Translational and Clinical Research Centers, Massachusetts General Hospital, Boston, MA, United States; 4 Sidney Kimmel Medical College, Thomas Jefferson University, Philadelphia, PA, United States; 5 Broad Institute of MIT and Harvard, Cambridge, MA, United States

**Keywords:** mediterranean diet, microbiome, randomized controlled trial, specific carbohydrate diet, ulcerative colitis

## Abstract

**Background and aims:**

This pilot randomized controlled-feeding trial compared the effect of Specific Carbohydrate Diet (SCD) and Mediterranean diet (MeD) in mild to moderate ulcerative colitis (UC).

**Methods:**

Seventeen adults were randomized to a 6-week SCD (*n* = 8) or MeD (*n* = 9) intervention. Primary outcome was change in partial Mayo Clinic score (pMCS).

**Results:**

The study was discontinued early due to significant dropout (*n* = 9, 52.9%). There was no significant between-group differences observed for pMCS change (SCD, −0.8; MeD, −1.3; *p* = 0.499) or secondary outcomes. Exploratory metagenomic analysis revealed enrichment of *Parasutterella excrementihominis* in SCD at week 10.

**Conclusion:**

In this pilot trial, SCD and MeD showed no difference in therapeutic effects for patients with mild to moderate UC. However, the study was limited by a significant drop out in both arms.

**Clinical trial registration:**

ClinicalTrials.gov, identifier NCT04398550.

## Introduction

Ulcerative colitis (UC) is a chronic inflammatory condition of the large intestine characterized by diffuse inflammation of the colonic mucosa (1). Although the exact mechanism underlying UC development is largely unknown, the gut microbiome is widely thought to play a crucial role in the pathogenesis and progression of the disease (1). Diet is a modifiable environmental factor that can shape the composition and function of the gut microbiome, influence intestinal barrier, and alter immune and inflammatory pathways relevant to UC (2–4).

Despite growing interest among patients and clinicians, robust evidence to guide dietary recommendations for UC remains limited, and current clinical guidance is largely based on expert opinion rather than randomized controlled trials (5). The Mediterranean diet (MeD), rich in fruits, vegetables, whole grains, fish, and olive oil, has been associated with reduced inflammation and improved quality of life among patients with UC (2, 6). The Specific Carbohydrate Diet (SCD), which excludes grains, refined sugars, and most dairy products (7), may attenuate inflammation in inflammatory bowel disease (IBD) by restoring microbial balance and enhancing mucosal integrity (8, 9), with preliminary studies showing symptomatic improvement in UC (10, 11).

A direct comparison of SCD and MeD is clinically relevant because both diets are commonly adopted by patients with IBD, yet they differ substantially in composition and ease of adherence. A prior randomized trial in Crohn’s disease found that SCD was not superior to MeD for symptomatic remission or inflammatory biomarker improvement (12). However, dietary and microbial responses may differ between Crohn’s disease and UC, underscoring the need for UC-specific randomized data (12, 13).

Therefore, we conducted a pilot randomized controlled-feeding trial comparing the effects of SCD and MeD on clinical, inflammatory, quality of life (QoL), and gut microbiome outcomes in adult patients with mild to moderate UC (5). The controlled-feeding design, where meals were prepared and provided to participants, allowed us to reduce variability in dietary intake and explore both the potential clinical effects and feasibility of these dietary interventions in patients with active UC.

## Materials and methods

This randomized, parallel-group, controlled-feeding trial compared the therapeutic effects of SCD and MeD in patients with mild to moderate UC. Patients were recruited from the Massachusetts General Hospital (MGH) Crohn’s and Colitis Center between September 2020 and April 2023. The trial followed CONSORT guidelines, received Mass General Brigham IRB approval (2020P000298), and was registered on ClinicalTrials.gov (NCT04398550).

Adults aged 18–75 years with confirmed UC and partial Mayo Clinic score (pMCS) 2–6 were eligible. Key exclusions were Crohn’s disease, severe or fulminant UC, prior colectomy or ostomy, and recent antibiotic use (see Supplementary material for a full list of exclusion criteria). Participants were randomized 1:1 to SCD or MeD. Investigators and participants were blinded to diet allocation; kitchen staff were unblinded for meal preparation. All meals and snacks were prepared by the MGH Metabolic Kitchen and provided for six weeks (Supplementary Table S1), with calories tailored to individual requirements (Supplementary Tables S2, S3). Weekly dietitian visits monitored weight, adherence, and adverse events. Participants were followed for an additional four weeks following the 6-week intervention.

The primary outcome was change in pMCS from baseline to week 6. Secondary outcomes included clinical remission (pMCS ≤ 1), fecal calprotectin (FC) ≤ 150 μg/g, C-reactive protein ≤ 5 mg/L, and quality of life scores (IBDQ-10 and SF-12). Outcomes were compared using analysis of covariance (ANCOVA) for continuous and logistic regression for categorical ones, adjusted for baseline values. All analyses were intention-to-treat. For missing week-6 outcomes, baseline observation carried forward (BOCF) was used in the intention-to-treat analysis, such that participants with missing week-6 data were assigned their baseline value. This approach assumes no improvement from baseline among participants with missing week-6 outcomes and was chosen as a conservative strategy to avoid overestimating treatment benefit (13).

Stool samples were collected at baseline, week 6, and week 10 for shotgun metagenomic sequencing (Illumina HiSeq 2,500, Broad Institute). Taxonomic and metabolic profiles were derived using MetaPhlAn 4 and HUMAnN 3 within the bioBakery workflow. Microbial associations with time were tested using MaAsLin 2 as an exploratory analysis (Supplementary material). False discovery rate correction (FDR) was applied (*q* < 0.25).

## Results

Of 38 screened individuals, 17 patients with mild to moderate UC were enrolled, with eight randomized to the SCD arm and nine to the MeD arm (Supplementary Figure S1). The mean age was 41.5 years, BMI 27.1 kg/m^2^, and 47% were male. Baseline disease activity was similar between arms (mean pMCS 4.2), though BMI was higher in the MeD arm (28.9 vs. 25.1) (Table 1).

**Table 1:** Baseline characteristics of patients in each diet arm.

Characteristics	SCD (*n* = 8)	MeD (*n* = 9)	SMD
Age (years), mean (SD)	38.4 (15.8)	44.2 (18.3)	0.342
BMI (kg/m^2^), mean (SD)	25.1 (4.6)	28.9 (4.5)	**0.842**
Male sex (%)	3 (37.5)	5 (55.6)	0.368
Race, *n* (%)			
White	7 (87.5)	8 (88.9)	0.043
Black/African American	1 (12.5)	1 (11.1)	
Hispanic, *n* (%)	1 (12.5)	0 (0.0)	0.535
Fecal calprotectin, *n* (%)			
> 150 μg/g	6/6 (100.0)	8/8 (100.0)	< 0.001
≤ 150 μg/g	0/6 (0)	0/8 (0)	
CRP, *n* (%)			
> 5 mg/L	3 (37.5)	3 (33.3)	0.087
≤ 5 mg/L	5 (62.5)	6 (66.7)	
SCCAI, mean (SD)	5.9 (2.2)	4.7 (2.5)	0.504
pMCS, mean (SD)	4.4 (1.3)	4.1 (1.8)	0.170
IBDQ-10, mean (SD)	46.6 (7.1)	50.2 (8.3)	0.467
SF-12 physical score, mean (SD)	45.3 (7.4)	49.4 (2.6)	0.72
SF-12 mental score, mean (SD)	48.5 (7.8)	48.3 (11.8)	0.018
IBD surgery, *n* (%)	0/6 (0.0)	0/8 (0.0)	< 0.001
Regular NSAID in past 2 years, *n* (%)	1/7 (14.3)	1/8 (12.5)	0.052
Mesalamine, *n* (%)	8 (100.0)	8 (88.9)	0.500
Steroids, *n* (%)	6 (75.0)	8 (88.9)	0.367
Immunosuppressives, *n* (%)	1 (12.5)	3 (33.3)	0.512
Biologics, *n* (%)	4 (50.0)	4 (44.4)	0.111

Bolded values indicate imbalance between arms.

SCD, specific carbohydrate diet; MeD, Mediterranean diet; SMD, standardized mean difference; SCCAI, simple clinical colitis activity index; pMCS, partial Mayo Clinic score; IBDQ-10, inflammatory bowel disease questionnaire-10; SF-12, 12-item short form health survey score; NSAID, nonsteroidal anti-inflammatory drug.

**Figure 1:**
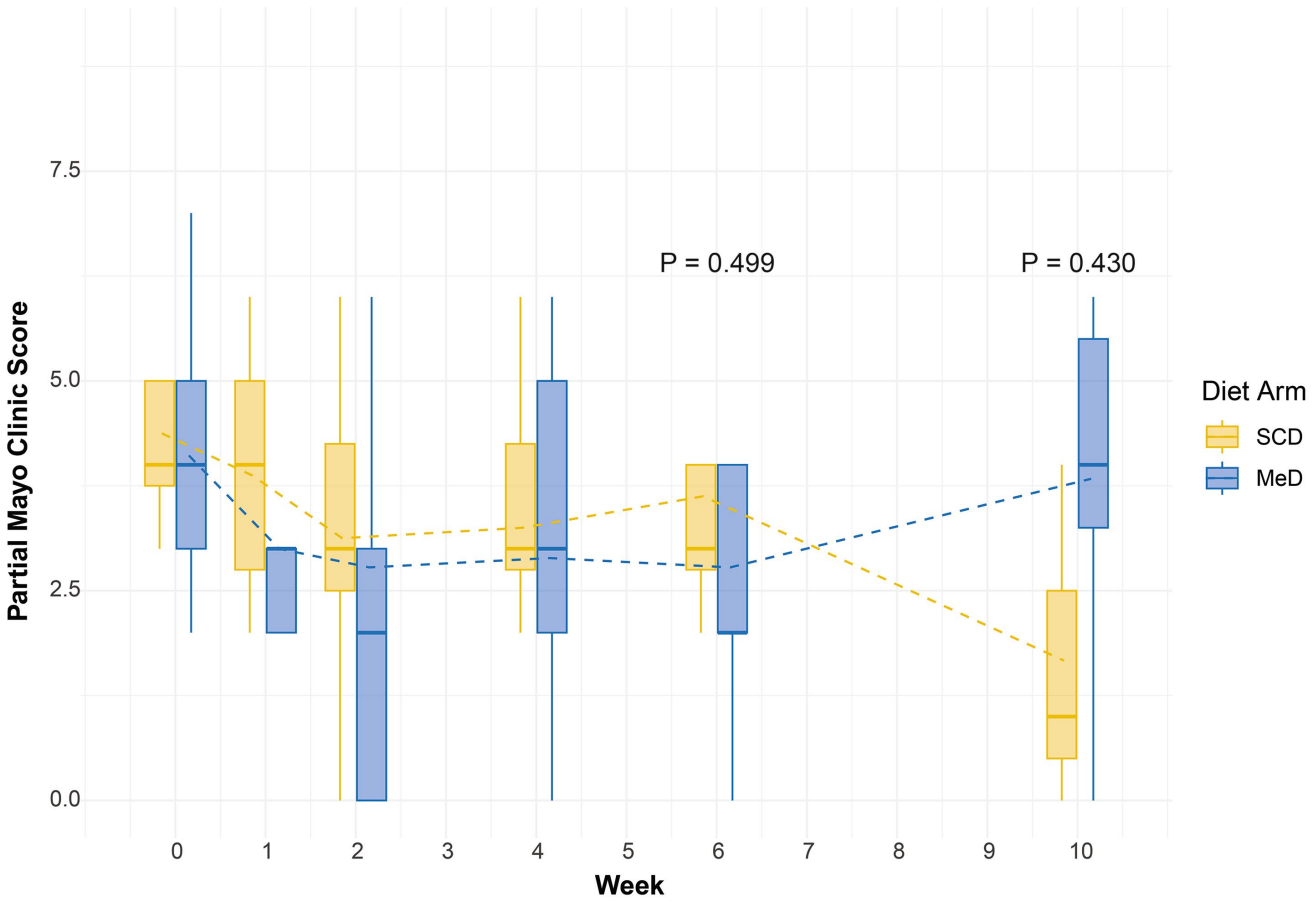
Partial Mayo Clinic score change over time across diet arms. From baseline to week 6, the mean pMCS decreases in similar magnitude in both arms, indicating improvement in symptoms over time. However, the treatment effects of MeD appear to wane by week 10. Boxes represent the interquartile range (25th to 75th percentiles), with horizontal lines indicating the median. Whiskers extend to 1.5 times the IQR from the lower and upper quartiles. Dashed lines connect group means across time points to illustrate longitudinal trends within each diet arm.

**Figure 2:**
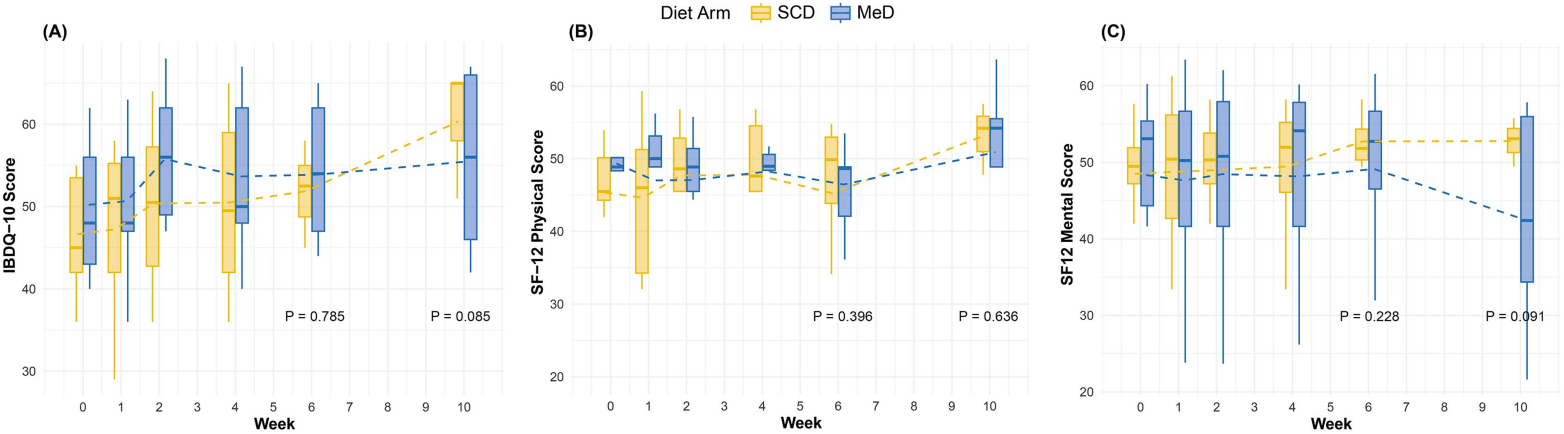
Secondary outcomes – IBDQ-10 and SF-12 physical and mental scores. Patients in both arms demonstrate similar improvements in IBD-related quality of life, as assessed by the IBDQ-10 score **(A)**, and general quality of life, as measured by the SF-12 physical score **(B)**. However, a divergent trend is observed in SF-12 mental scores, with patients on the MeD showing a decreasing trend at week 10 **(C)**. Despite these trends, the differences between the diet arms are not statistically significant.

From baseline to week 6, overall mean pMCS decreased from 4.2 to 3.2 (*p* = 0.026). Within arms, raw mean pMCS declined from 4.4 to 3.6 on SCD (*p* = 0.080) and from 4.1 to 2.8 on MeD (*p* = 0.119). In analysis adjusted for baseline pMCS, the week-6 mean pMCS was 3.51 for SCD and 2.88 for MeD, corresponding to a mean difference of 0.63 for SCD versus MeD (95% CI, −1.32 to 2.57; *p* = 0.499) (Figure 1). By week 10, pMCS changes remained comparable (adjusted mean difference: -1.58; 95% CI, −6.16 to 2.99; *p* = 0.430). Clinical remission occurred in 0% on SCD and 22.2% on MeD (*p* = 0.471) (Supplementary Table S4). No significant between-group differences were observed in FC (16.7% vs. 37.5%; *p* = 0.404) or CRP normalization (50.0% vs. 66.7%; *p* = 0.998). Mean weight change at week 6 was −2.3 kg on SCD and −1.8 kg on MeD (*p* = 0.724). Quality of life improved across all participants: mean IBDQ-10 increased from 48.5 to 52.9 at week 6 (*p* = 0.009) and to 57.1 at week 10 (*p* = 0.004). When adjusting for baseline values, there was no significant between-arm difference at week 6 (adjusted mean difference: 0.83; 95% CI, −5.55 to 7.20; *p* = 0.785) or week 10 (adjusted mean difference: 5.61; 95% CI, −1.05 to 12.30; *p* = 0.085). SF-12 physical and mental scores did not change significantly at week 6 (physical score adjusted mean difference: 3.29; 95% CI, −4.86 to 11.40; *p* = 0.396) (mental score adjusted mean difference: 2.83; 95% CI, −2.03 to 7.68; *p* = 0.228) or week 10 (physical score adjusted mean difference: -2.49; 95% CI, −16 to 11; *p* = 0.636) (mental score adjusted mean difference: 11.7; 95% CI, −2.98 to 26.4; *p* = 0.091) (Figures 2A–C).

Five participants (62.5%) from the SCD arm and four (44.4%) from the MeD arm withdrew before week 6, mostly due to dietary intolerance or worsening disease (*p* = 0.637) (Supplementary Figure S1). Fourteen of 17 (82.4%) adhered fully to assigned diets (Supplementary Table S5). No severe adverse events occurred (Supplementary Table S6). Mild gastrointestinal symptoms were reported in one SCD and three MeD participants (*p* = 0.661).

Thirty-eight stool samples [Baseline: *n* = 14 (SCD = 6, MeD = 8); Week 6: *n* = 14 (SCD = 7, MeD = 7); Week 10: *n* = 6 (SCD = 2, MeD = 4)] were available for metagenomic sequencing. Microbiome alpha diversity did not differ between arms or across timepoints, while principal coordinate analysis showed moderate between-group distinctions (Supplementary Figures S2A,B). Diet assignment over time and weeks since diet initiation explained 4.2 and 3.6% of overall variance in microbiome composition, respectively, but were not statistically significant (Supplementary Figure S3). In the exploratory metagenomic analysis, *Parasutterella excrementihominis* was enriched at week 10 in the SCD arm (*q* = 0.243), whereas no significant taxonomic or pathway changes were observed in the MeD arm.

## Discussion

In this randomized controlled-feeding trial of adults with mild to moderate UC, reductions in pMCS were comparable between the SCD and MeD. Secondary outcomes, including clinical remission, inflammatory markers, and quality of life, were also similar. The study was discontinued early on January 8th, 2024 due to limited efficacy and high dropout rates related to diet intolerance or worsening disease. However, the high drop out in our study highlighted the feasibility concerns of conducting feeding studies of these two diets in patients with UC and severely limited our ability to compare their efficacy in mild to moderately active UC.

Our findings are largely consistent with prior clinical trials of diet in UC, which have shown heterogenous responses with little to modest subjective or objective improvements (14–16). The high withdrawal rate in our trial further highlights the feasibility and tolerability challenges of implementing controlled-feeding dietary interventions in patients with active UC. Collectively, these findings underscore the importance of feasibility, palatability, patient preference, and retention strategies when designing future dietary intervention trials in IBD.

Strengths of our study include the controlled-feeding design and metagenomic sequencing, which allowed us to reduce variability in dietary intake and explore potential microbiome changes. Limitations include small sample size, short intervention, early discontinuation, high dropout rate, and reliance on self-reported adherence.

In conclusion, in this pilot randomized controlled-feeding study, we did not observe significant differences between SCD and MeD diets in reducing disease activity, lowering inflammatory markers, or improving QoL in patients with mild to moderate UC. We also observed a high drop out and significant issues with tolerability of diets in patients with active UC. Therefore, the lack of observed between-group differences in this pilot trial should not be interpreted as evidence that either diet is ineffective, but rather as preliminary data to inform the design of larger trials. Larger, adequately powered studies with strategies to improve tolerability are needed before firm conclusions can be made regarding the comparative efficacy of SCD and MeD in UC.

## Data Availability

The datasets presented in this article are not readily available because patient data are under HIPPA protection. Requests to access the datasets should be directed to Hamed Khalili (hkhalili@mgh.harvard.edu).

## References

[1] Sartor RB. Mechanisms of disease: pathogenesis of Crohn's disease and ulcerative colitis. Nat Clin Pract Gastroenterol Hepatol. (2006) 3:390–407. doi: 10.1038/ncpgasthep0528, 16819502

[2] De F F P N V L Bj I La S A L L . High-level adherence to a Mediterranean diet beneficially impacts the gut microbiota and associated metabolome. Gut. (2016) 65:1812. doi: 10.1136/gutjnl-2015-309957 26416813

[3] Honda K Littman DR. The microbiota in adaptive immune homeostasis and disease. Nature. (2016) 535:75–84. doi: 10.1038/nature18848, 27383982

[4] Khor B Gardet A Xavier RJ. Genetics and pathogenesis of inflammatory bowel disease. Nature. (2011) 474:307–17. doi: 10.1038/nature10209, 21677747 PMC3204665

[5] Hashash JG Elkins J Lewis JD Binion DG. AGA clinical practice update on diet and nutritional therapies in patients with inflammatory bowel disease: expert review. Gastroenterology. (2024) 166:521–32. doi: 10.1053/j.gastro.2023.11.303, 38276922

[6] Chicco F Magrì S Cingolani A Paduano D Pesenti M Zara F . Multidimensional impact of Mediterranean diet on IBD patients. Inflamm Bowel Dis. (2020) 27:1–9. doi: 10.1093/ibd/izaa097, 32440680 PMC7737160

[7] Suskind DL Wahbeh G Gregory N Vendettuoli H Christie D. Nutritional therapy in pediatric Crohn disease: the specific carbohydrate diet. J Pediatr Gastroenterol Nutr. (2014) 58:87–91. doi: 10.1097/MPG.0000000000000103, 24048168

[8] Suskind DL Lee D Kim Y-M Wahbeh G Singh N Braly K . The specific carbohydrate diet and diet modification as induction therapy for pediatric Crohn’s disease: a randomized diet controlled trial. Nutrients. (2020) 12:3749. doi: 10.3390/nu12123749, 33291229 PMC7762109

[9] Lindsay JO Whelan K Stagg AJ Gobin P Al-Hassi HO Rayment N . Clinical, microbiological, and immunological effects of fructo-oligosaccharide in patients with Crohn’s disease. Gut. (2006) 55:348–55. doi: 10.1136/gut.2005.074971, 16162680 PMC1856087

[10] Obih C Wahbeh G Lee D Braly K Giefer M Shaffer ML . Specific carbohydrate diet for pediatric inflammatory bowel disease in clinical practice within an academic IBD center. Nutrition. (2016) 32:418–25. doi: 10.1016/j.nut.2015.08.025 26655069

[11] Suskind DL Wahbeh G Cohen SA Damman CJ Klein J Braly K . Patients perceive clinical benefit with the specific carbohydrate diet for inflammatory bowel disease. Dig Dis Sci. (2016) 61:3255–60. doi: 10.1007/s10620-016-4307-y, 27638834

[12] Lewis JD Sandler RS Brotherton C Brensinger C Li H Kappelman MD . A randomized trial comparing the specific carbohydrate diet to a Mediterranean diet in adults with Crohn’s disease. Gastroenterology. (2021) 161:837–852.e9. doi: 10.1053/j.gastro.2021.05.047, 34052278 PMC8396394

[13] Kaiser KA Affuso O Beasley TM Allison DB. Getting carried away: a note showing baseline observation carried forward (BOCF) results can be calculated from published complete-cases results. Int J Obes. (2012) 36:886–9. doi: 10.1038/ijo.2011.25, 21407169 PMC3130885

[14] Kaplan HC Opipari-Arrigan L Yang J Schmid CH Schuler CL Saeed SA . Personalized research on diet in ulcerative colitis and Crohn's disease: a series of N-of-1 diet trials. Official J American College Gastroenterol. (2022) 117:902–917. doi: 10.14309/ajg.0000000000001800 35442220

[15] Sarbagili-Shabat C Albenberg L Van Limbergen J Pressman N Otley A Yaakov M . A novel UC exclusion diet and antibiotics for treatment of mild to moderate pediatric ulcerative colitis: a prospective open-label pilot study. Nutrients. (2021) 13:3736. doi: 10.3390/nu13113736, 34835992 PMC8622458

[16] Fritsch J Garces L Quintero MA Pignac-Kobinger J Santander AM Fernández I . Low-fat, high-Fiber diet reduces markers of inflammation and Dysbiosis and improves quality of life in patients with ulcerative colitis. Clin Gastroenterol Hepatol. (2021) 19:1189–1199.e30. doi: 10.1016/j.cgh.2020.05.026, 32445952

