# Supplementary material for: Specific carbohydrate diet versus Mediterranean diet in adult patients with mild to moderate ulcerative colitis: a randomized controlled-feeding trial
Source: Front Nutr. 2026 Jul 10;13:1838160. doi: 10.3389/fnut.2026.1838160 (PMC13395713; doi:10.3389/fnut.2026.1838160)
Supplement: Supplementary file 1 [file Supplementary_file_1.docx]

**Supplementary Methods**

***Study design***

This randomized, parallel-group, controlled-feeding trial compared SCD and MeD in mild to moderate UC. Patients were recruited from the MGH Crohn’s and Colitis Center (September 3, 2020 – April 19, 2023). The trial followed CONSORT, was approved by Mass General Brigham IRB (2020P000298), and registered (NCT04398550). All authors had access to data and approved the final manuscript.

***Patient eligibility***

Adults aged 18 to 75 years with a confirmed diagnosis of UC via colonoscopy or flexible sigmoidoscopy, and with active disease for at least three months prior to screening, were considered for inclusion. Eligible patients presented with mild to moderate symptoms, characterized by a partial Mayo Clinic score (pMCS) between 2 and 6 at screening (1). Additional criteria included an endoscopy subscore between 1 and 2 for those who have received a colonoscopy within the screening period, or a FC level exceeding 150 µg/g within four weeks of screening for those without a clinically indicated colonoscopy. Patients receiving 5-aminosalicylates (5-ASAs) had to be on a stable dose for at least four weeks before screening. Those on immunosuppressive therapies or biologic agents required a stable dose for eight weeks before baseline. At baseline, corticosteroid use was permitted but limited to a maximum of 20 mg of prednisone or 9 mg of budesonide MMX.

Exclusion criteria included diagnosis of CD, intermediate colitis, severe to fulminant colitis, hemoglobin < 8.0 g/dL or albumin < 3.0 g/dL, a history of colectomy, colonic dysplasia, presence of an ileal pouch or ostomy, or active bacterial or viral gastroenteritis. Patients who had been hospitalized for UC requiring intravenous steroids or had received systemic antibiotics within two weeks before screening were also ineligible. Other exclusion factors included the use of total parenteral nutrition, anti-diarrheal medications, or recent treatment with cyclosporine, tacrolimus, or thalidomide within two months before screening, or a history of non-compliance or dietary preferences that could interfere with adherence to study procedures.

***Randomization and dietary intervention***

Eligible patients were assigned in a 1:1 ratio to either the SCD or MeD according to a computer-generated randomization system. The investigators and patients were blinded to the allocation of diet. Because meals were prepared by the metabolic kitchen at the Metabolism and Nutrition Research Center at MGH Translational and Clinical Research Centers (TCRC), TCRC staff were necessarily unblinded. The investigators, participants, and all other study staff were not intentionally unblinded. However, given the nature of the interventions, participants and study personnel who interacted with them could infer the assigned diet, though the allocation was never explicitly disclosed.

For the first six weeks, patients received three meals and two snacks per day prepared by the TCRC metabolic kitchen. Foods were picked up every 5-7 or 12-14 days, and patients were instructed to purchase selected perishable items independently with reimbursement. Pre-defined three-day cycle menus for both diets were developed in Nutrition Data System for Research software version 2019 (NCC University of Minnesota, Minneapolis, MN) and are provided in the **Table S1**. Total calories from the provided meals were tailored according to each patient’s energy requirements (**Table S2 and S3**). Once a week, coinciding with food pick-up, patients met with a dietitian at the TCRC to monitor weight and adherence and to review any concerns. After the six-week dietary intervention, patients were followed for an additional four weeks. Patients were allowed to withdraw from the trial at any time. Those withdrawn early were asked to complete an early discontinuation visit where they were offered the opportunity to have their blood, stool, and questionnaires collected at that time.

***Outcome measurements***

The primary outcome of this trial is change in the clinical activity score – pMCS after 6 weeks of dietary intervention (1). The secondary outcomes measured at week 6 were proportion of clinical remission (pMCS ≤ 1), FC response (reduction of FC to ≤ 150 µg/g), and normal CRP level (CRP ≤ 5 mg/L). The secondary outcomes measured at both week 6 and 10 were QoL measurements, including the short inflammatory bowel disease questionnaire (IBDQ-10) (2) and the 12-Item Short Form Health Survey (SF-12) scores (3). The raw SF-12 physical score and SF-12 mental score were weighted and transformed according to the SF-12 manual (3). For all outcomes, missing data up to week 6 were imputed using the Baseline Observation Carried Forward (BOCF) method (4, 5). This approach assumes no improvement from baseline among participants with missing week-6 outcomes and was chosen as a conservative strategy to avoid overestimating treatment benefit.

***Diet adherence assessment and adverse event monitoring***

At the end of follow-up, adherence to the assigned diets during the full 6-week intervention was evaluated based on participant-completed daily logs recording the percentage of each food item the participant consumed. Adherence was categorized using the following scale: *Sometimes* (≤ 75% of the time), and *always* (> 75% of the time) following the diet. An adverse event was defined as any untoward medical occurrence temporally associated with the diet, including unfavorable signs (e.g., abnormal laboratory findings), symptoms, or new/exacerbated diseases. All adverse events were reported to the study principal investigator within 24 hours.

***Stool microbiome analysis***

Consented patients were provided with a stool collection kit for FC and microbiome analysis prior to their scheduled intake appointment. Patients were instructed to collect their stool sample 1-2 days before their scheduled visit and brought the specimens at the time of their appointment or shipped the specimens to our center at ambient temperature. The stool samples were collected per the standard protocols from Human Microbiome Project (HMP) (6) at three study timepoints: baseline, week 6, and week 10. Samples were preserved in 95% ethanol at room temperature for less than 48 hours before being stored at -80ºC. All samples were analyzed in a single batch at the completion of this study. Stool genomic DNA purification was performed based on the QIAGEN AllPrep MiniKit (Valencia, CA, USA) kit. Purified DNA were processed using Nextera XT DNA Library Preparation Kit (Illumina, San Diego, CA, USA) per the manufacturer’s protocol to generate metagenomic sequencing libraries. Subsequently, shotgun metagenomic sequencing was performed at the Broad Institute (Cambridge, MA, USA) utilizing the Illumina HiSeq 2500 platform. The initial processing of raw metagenomic reads was performed with KneadData (v0.12.0), eliminating Illumina adapter sequences and reads shorter than 50 base pairs. Human-derived reads were filtered using Bowtie2 (v2.5.1) with the Genome Reference Consortium human genome (GRCh37/hg19) as a reference. Quality assessment was conducted with FastQC (v0.12.0). During this process, four samples with more than 90% reads lost after decontamination were excluded. Finally, 34 samples (out of 38 samples, 89.5%) were included in the microbiome analysis, with a post-quality control mean read depth of 38.2 ± 18.8 million (~ 5.7 Gbp per sample).

We profiled the taxonomic composition and microbial functional potentials by analyzing metagenomics through the bioBakery meta'omics workflow (7). We applied the Metagenomic Phylogenetic Analysis tool (MetaPhlAn v4.0.6) to align the sample shotgun sequences against the tool’s database to characterize species relative abundance in each sample (8). The HMP Unified Metabolic Analysis Network (HUMAnN3 v3.8) (9) was used to compare the metagenomic sequences to two databases, the ChocoPhlAn pangenome (10) and the UniRef90 protein (11), to reconstruct the microbial metabolic pathways. For downstream analyses, we only assessed taxonomic species with relative abundance ≥ 0.01% and metabolic pathways with relative abundance ≥ 0.1%, which were present in more than 10% of samples (12).

***Statistical analysis***

The target sample size was determined based on the primary outcome. To detect a mean difference of at least 0.8 in pMCS between the SCD and MeD arms with 80% power and a 5% type I error rate, a total of 50 patients was required, with 25 allocated to each arm. We compared continuous outcomes, including pMCS, IBDQ-10, and SF-12 scores at week 6 and week 10 according to their treatment allocation while accounting for their baseline scores using analysis of covariance (ANCOVA). The dichotomous outcomes including proportion of clinical remission, FC response, and normal CRP level at week 6 were compared by logistic regression with their baseline as an independent variable. All analyses were conducted as intention-to-treat. Standardized mean differences (SMDs) were used to compare the baseline characteristics between diet arms. An SMD less than 0.49 was interpreted as small, 0.50 to 0.79 as medium, and greater than 0.79 as large (13).

From the metagenomic output, we compared the alpha diversity (Shannon index) in both arms at baseline, week 6, and week 10 using the Wilcoxon rank-sum test. To evaluate the difference in microbiome composition between both arms, we calculated the Bray-Curtis dissimilarity and generated a principal coordinates analysis (PCoA) plot by applying the classical multidimensional scaling (14). The variation in microbiome composition attributable treatment arms and patient characteristics, including age at enrollment, sex, and body mass index (BMI), was quantified using permutational multivariate analysis of variance (PERMANOVA) with 999 permutations. All analyses were conducted in R using the Vegan package (v2.6-4) (15).

To evaluate temporal changes in the relative abundance of taxonomic composition and metabolic pathways in the gut microbiome across different dietary interventions, we performed separate exploratory analyses for each dietary arm using the Microbiome Multivariable Association with Linear Models (MaAsLin2 v3.18, <http://huttenhower.sph.harvard.edu/maaslin2>) (16). The models were adjusted for key covariates, including age at enrollment, sex, and BMI. Age and BMI were standardized into z-scores to enhance comparability. To account for within-subject variability, patient IDs were incorporated as random effects. The linear model was specified as:

*Log (Microbiome features / Microbial metabolic pathways) ~ Timepoint (week 6 vs week 10 vs baseline) + age + sex + BMI + (1 | patient_ID)*

In all analyses, two-sided p-values below 0.05 were considered statistically significant. When appropriate, multiple testing correction was performed using the Benjamini-Hochberg method, with statistical significance defined as a false discovery rate (FDR) q-value < 0.25. This threshold aligns with previous studies in microbiome research (17). All p-values reported were two-sided.

**Table S1: Three-day cycle menus for the SCD and MeD diet**

**The SCD menu**

| **Cycle 1:**  ***Breakfast*** | ***Lunch*** | ***Dinner*** |
| --- | --- | --- |
| - Fresh apple - Homemade nut muffin with butter - Whole milk lactose-free plain yogurt - Honey | - Tuna sandwich* on homemade cheese bread - Cheddar Cheese slices - Baby carrots - Homemade Granola Chew - Fresh Clementine   *No fish option:  substitute hard boiled eggs for tuna salad | - Spiralized carrots with tomato sauce with meat - Peas with butter - Whole milk lactose-free plain yogurt   ***Snacks***   - Cheddar cheese - Fresh apple |
| **Cycle 2:**  ***Breakfast*** | ***Lunch*** | ***Dinner*** |
| - Nut waffle with butter and honey - Whole milk lactose-free plain yogurt - Apple juice | - Cheeseburger with lettuce on homemade cheese bread - Pineapple chunks - Whole milk lactose-free plain yogurt | - Beef chili - Spinach - Homemade cheese bread - Peanut Butter cookie     ***Snacks***   - Fresh apple with peanut butter |
| **Cycle 3:**  ***Breakfast*** | ***Lunch*** | ***Dinner*** |
| - Cashew cookie breakfast bar - Whole milk lactose-free plain yogurt - Fresh Apple/ Peanut butter - Grape juice | - Mixed greens salad with cheddar cheese - Olive oil and vinegar dressing - Homemade cheese bread with butter | - Fish Casserole - Whole milk lactose-free plain yogurt - Spinach and carrots with butter - Pineapple juice/orange juice mix   *No fish option:   - Hamburger Pie - Whole milk lactose-free plain yogurt - Spinach and carrots - Fresh clementine     ***Snacks***   - Almonds and raisins |

Note: Baked goods for SCD were grain-free.

**The MeD menu**

| **Cycle 1:**  ***Breakfast*** | ***Lunch*** | ***Dinner*** |
| --- | --- | --- |
| - Almond/Coconut breakfast bar - Fresh banana - Low fat plain yogurt | - Tuna sandwich* with lettuce on whole wheat bread - Mayonnaise / mustard - Fresh orange - Multigrain chips - Guacamole   *No fish option:  Grilled chicken sandwich | - Lentil soup - Scallion rice - Broccoli - Pineapple chunks - Low fat plain yogurt   ***Snacks***   - Walnuts - Dried apricots |
| **Cycle 2:**  ***Breakfast*** | ***Lunch*** | ***Dinner*** |
| - Whole grain cereal - 1% Milk - Whole wheat bagel - Peanut butter - Fresh banana | - Black bean burger - Whole wheat roll - Lettuce / tomato - Mayonnaise - Low fat plain yogurt | - Salmon* - Quinoa pilaf - Spinach - Cauliflower - Low fat plain yogurt - Orange Juice   *No fish option:  Grilled chicken  ***Snacks***   - Hummus - Whole grain Crackers - Almonds |
| **Cycle 3:**  ***Breakfast*** | ***Lunch*** | ***Dinner*** |
| - Banana nut muffin - Fresh banana / Peanut butter - Low fat plain yogurt | - Mixed greens salad with black / Garbanzo beans - Olive oil and vinegar dressing - Whole wheat bread - Soy milk | - Baked Cod* - Quinoa pilaf - Butternut squash - Spinach - Pineapple chunks   *No fish option:  Grilled chicken  ***Snacks***   - Low fat plain yogurt - Walnuts - Dried apricots |

**Table S2: Nutrient summary for the SCD and MeD diet**

| **SCD** | **1100 kcals** | **1350 kcals** | **1600 kcals** | **2100 kcals** | **2600 kcals** | **3100 kcals** | **Average** |
| --- | --- | --- | --- | --- | --- | --- | --- |
| **Energy (kilocalories)** | 1103 |  | 1598 | 2105 | 2604 | 3099 | 2101.80 |
| **Total Fat (g)** | 64.597 |  | 95.661 | 127.016 | 156.397 | 190.338 | 126.80 |
| **Total Carbohydrate (g)** | 88.086 |  | 126.651 | 162.258 | 200.443 | 233.748 | 162.24 |
| **Total Protein (g)** | 53.115 |  | 73.167 | 97.702 | 122.535 | 142.265 | 97.76 |
| **Animal Protein (g)** | 41.013 |  | 54.639 | 73.406 | 93.535 | 105.716 | 73.66 |
| **Vegetable Protein (g)** | 12.102 |  | 18.529 | 24.296 | 29.000 | 36.549 | 24.10 |
| **% Calories from Fat** | 51.09% |  | 52.10% | 52.54% | 52.30% | 53.43% | 52.29% |
| **% Calories from Carbohydrate** | 29.75% |  | 29.71% | 28.99% | 28.92% | 28.30% | 29.13% |
| **% Calories from Protein** | 19.16% |  | 18.18% | 18.47% | 18.78% | 18.26% | 18.57% |
| **Cholesterol (mg)** | 225 |  | 319 | 426 | 528 | 637 | 427.00 |
| **Omega-3 Fatty Acids (g)** | 0.847 |  | 1.138 | 1.458 | 1.826 | 1.987 | 1.45 |
| **Total Dietary Fiber (g)** | 13.467 |  | 18.359 | 22.191 | 25.797 | 32.164 | 22.40 |
| **Calcium (mg)** | 756 |  | 1032 | 1378 | 1738 | 1943 | 1369.40 |
| **Iron (mg)** | 5.581 |  | 7.896 | 10.263 | 12.485 | 15.138 | 10.27 |
| **Zinc (mg)** | 8.150 |  | 11.179 | 14.918 | 18.919 | 22.118 | 15.06 |
| **Sodium (mg)** | 1138 |  | 1543 | 2077 | 2571 | 3017 | 2069.20 |
| **Potassium (mg)** | 1677 |  | 2415 | 3111 | 3797 | 4446 | 3089.20 |

| **MeD** | **1100 kcals** | **1350 kcals** | **1600 kcals** | **2100 kcals** | **2600 kcals** | **3100 kcals** | **Average** |
| --- | --- | --- | --- | --- | --- | --- | --- |
| **Energy (kilocalories)** | 1098 | 1340 | 1599 | 2095 | 2597 | 3096 | 1970.83 |
| **Total Fat (g)** | 43.124 | 56.000 | 64.844 | 84.793 | 101.904 | 131.589 | 80.38 |
| **Total Carbohydrate (g)** | 142.803 | 166.000 | 201.437 | 264.474 | 331.055 | 379.899 | 247.61 |
| **Total Protein (g)** | 48.226 | 58.960 | 70.545 | 92.265 | 116.869 | 131.805 | 86.45 |
| **Animal Protein (g)** | 16.824 | 17.580 | 26.496 | 34.583 | 46.124 | 51.298 | 32.15 |
| **Vegetable Protein (g)** | 31.403 | 41.379 | 44.050 | 57.682 | 70.746 | 80.508 | 54.29 |
| **% Calories from Fat** | 33.60% | 35.87% | 34.94% | 34.73% | 33.71% | 36.55% | 34.90% |
| **% Calories from Carbohydrate** | 50.07% | 48.04% | 48.56% | 48.74% | 49.25% | 47.36% | 48.67% |
| **% Calories from Protein** | 16.29% | 16.10% | 16.47% | 16.51% | 17.01% | 16.06% | 16.41% |
| **Cholesterol (mg)** | 24 | 30 | 45 | 52 | 61 | 80 | 48.67 |
| **Omega-3 Fatty Acids (g)** | 2.666 | 3.280 | 3.930 | 4.775 | 5.903 | 7.186 | 4.62 |
| **Total Dietary Fiber (g)** | 25.041 | 31.800 | 35.004 | 43.370 | 52.183 | 61.697 | 41.52 |
| **Calcium (mg)** | 695 | 755 | 1034 | 1460 | 1910 | 2034 | 1314.67 |
| **Iron (mg)** | 9.961 | 12.874 | 14.130 | 19.275 | 23.897 | 26.554 | 17.78 |
| **Zinc (mg)** | 6.517 | 7.683 | 9.343 | 12.907 | 16.694 | 18.262 | 11.90 |
| **Sodium (mg)** | 951 | 1349 | 1556 | 1949 | 2408 | 2895 | 1851.33 |
| **Potassium (mg)** | 2441 | 2965 | 3615 | 4551 | 5683 | 6551 | 4301.00 |

**Table S3: Nutrient summary for the no-fish version of SCD and MeD diet**

|  | **SCD fish** | **SCD no fish** | **% change** | **MeD fish** | **MeD no fish** | **% change** |
| --- | --- | --- | --- | --- | --- | --- |
| **Energy (kilocalories)** | 2105 | 2101 | -0.19% | 2095 | 2104 | 0.43% |
| **Total Fat (g)** | 127.016 | 131.387 | 3.44% | 84.793 | 83.328 | -1.73% |
| **Total Carbohydrate (g)** | 162.258 | 156.377 | -3.62% | 264.474 | 265.750 | 0.48% |
| **Total Protein (g)** | 97.702 | 92.096 | -5.74% | 92.265 | 95.762 | 3.79% |
| **Animal Protein (g)** | 73.406 | 68.602 | -6.54% | 34.583 | 38.090 | 10.14% |
| **Animal Protein (%)** | 75.13% | 74.49% | -0.86% | 37.48% | 39.78% | 6.12% |
| **Vegetable Protein (g)** | 24.296 | 23.494 | -3.30% | 57.682 | 57.673 | -0.02% |
| **Animal Protein (%)** | 24.87% | 25.51% | 2.59% | 62.52% | 60.23% | -3.67% |
| **% Calories from Fat** | 52.54% | 54.54% | 3.80% | 34.73% | 33.98% | -2.16% |
| **% Calories from Carbohydrate** | 28.99% | 27.95% | -3.60% | 48.74% | 48.80% | 0.11% |
| **% Calories from Protein** | 18.47% | 17.50% | -5.25% | 16.51% | 17.20% | 4.20% |
| **Cholesterol (mg)** | 426 | 582 | 36.62% | 52 | 64 | 23.08% |
| **Omega-3 Fatty Acids (g)** | 1.458 | 0.576 | -60.49% | 4.775 | 4.244 | -11.12% |
| **Total Dietary Fiber (g)** | 22.191 | 21.270 | -4.15% | 43.370 | 43.125 | -0.56% |
| **Calcium (mg)** | 1378 | 1249 | -9.36% | 1460 | 1450 | -0.68% |
| **Iron (mg)** | 10.263 | 10.775 | 4.99% | 19.275 | 19.390 | 0.60% |
| **Zinc (mg)** | 14.918 | 16.499 | 10.60% | 12.907 | 13.254 | 2.69% |
| **Sodium (mg)** | 2077 | 2086 | 0.43% | 1949 | 1922 | -1.39% |
| **Potassium (mg)** | 3111 | 2962 | -4.79% | 4551 | 4439 | -2.46% |

**Figure S1: Study enrollment, randomization to diet arms, and follow-up**

**
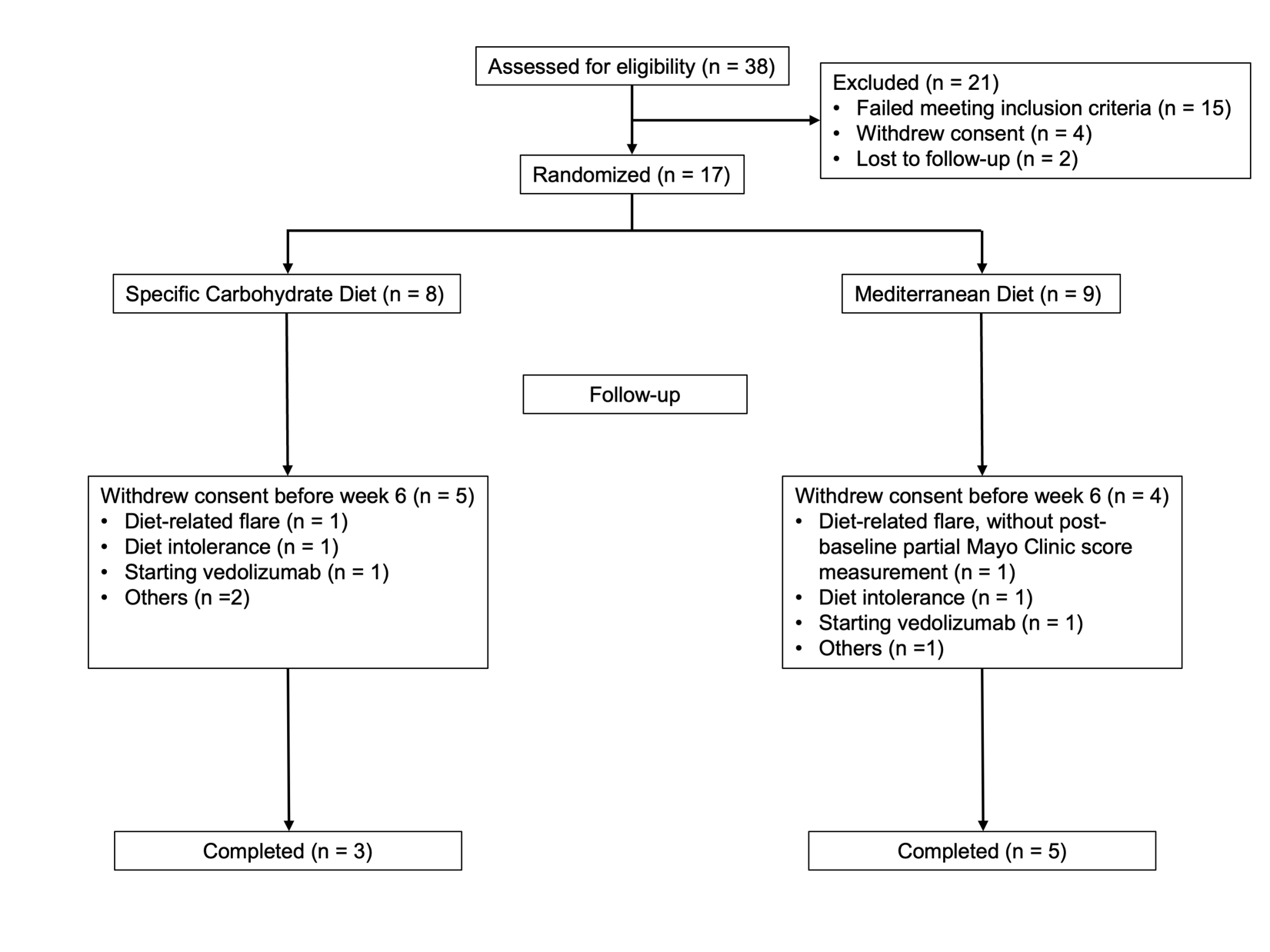
**

|  | **SCD** | **MeD** | **P-value** |
| --- | --- | --- | --- |
| **Clinical remission (pMCS ≤ 1)** | n = 8 | n = 9 | 0.471 |
| week 0 | 0 (0%) | 0 (0%) |  |
| week 6 | 0 (0%) | 2 (22.2%) |  |
| **FC response (FC ≤ 150 µg/g)** | n = 6 | n = 8 | 0.404 |
| week 0 | 0 (0%) | 0 (0%) |  |
| week 6 | 1 (16.7%) | 3 (37.5%) |  |
| **Normal CRP level (CRP ≤ 5 mg/L)** | n = 8 | n = 9 | 0.998 |
| week 0 | 5 (62.5%) | 6 (66.7%) |  |
| week 6 | 4 (50.0%) | 6 (66.7%) |  |
| Abbreviations: fecal calprotectin (FC), C-reactive protein (CRP) | | | |

**Table S4: Secondary outcomes – clinical remission, FC response, and normal CRP level.**

**Table S5: Patient adherence to assigned diet**

| **ID** | **Diet** | **wk1** | **wk2** | **wk3** | **wk4** | **wk5** | **wk6** | **Withdrawal time** |
| --- | --- | --- | --- | --- | --- | --- | --- | --- |
| **1** | **MeD** | Always | Always | Always | Always | Always | Always |  |
| **2** | **SCD** | Always | Always | Always | Always | Always |  | wk5 |
| **4** | **MeD** | Always | Always | Always | Always | Always | Always |  |
| **7** | **SCD** | Always |  |  |  |  |  | wk1 |
| **8** | **MeD** | Always | Always | Always | Always | Always | Always |  |
| **9** | **SCD** | Sometimes |  |  |  |  |  | wk2 |
| **11** | **MeD** | Always | Always | Always |  |  |  | wk3 |
| **12** | **MeD** | Always | Always |  |  |  |  | wk2 |
| **13** | **SCD** | Always | Always | Always | Always | Always |  | wk5 |
| **17** | **MeD** | Always | Always | Always | Always | Always | Always |  |
| **19** | **MeD** | NA |  |  |  |  |  | wk1 |
| **20** | **SCD** | Always | Always | Always | Always | Always | Always |  |
| **25** | **MeD** | Always | Always | Always | Always | Always | Always |  |
| **27** | **SCD** | Always | Always | Always | Always | Always | Always |  |
| **33** | **MeD** | Always | Always | Always |  |  |  | wk3 |
| **34** | **SCD** | Always | Sometimes | Always | Always | Always | Sometimes |  |
| **38** | **SCD** | Always |  |  |  |  |  | wk1 |

| **Type of adverse event** | **By week 6** | | **P-value** |
| --- | --- | --- | --- |
|  | **SCD,**  **n (%)** | **MeD,**  **n (%)** |  |
| **Gastrointestinal** | 1 (12.5) | 3 (33.3) | 0.661 |
| **Musculoskeletal** | 0 | 1 (11.1) | 1.000 |
| **Infection or**  **immune-related** | 0 | 1 (11.1) | 1.000 |
| **Rheumatologic** | 1 (12.5) | 0 | 0.952 |
| **Other** | 1 (12.5) | 2 (22.2) | 0.600 |
| No new adverse events were reported during week 6 to week 10. | | | |

**Table S6: Adverse events by week 6**

**Figure S2: Microbiome composition across diet arms**

(A) Alpha diversity, assessed by the Shannon index, demonstrates no significant differences between the SCD and MeD arms at baseline, week 6, or week 10. Additionally, there are no significant longitudinal changes in diversity within either dietary arm over time.

(B) Principal coordinate analysis (PCoA) based on Bray-Curtis dissimilarity shows overlapping clusters between the SCD and MeD groups, suggesting similar overall gut microbiome composition across the two diets.


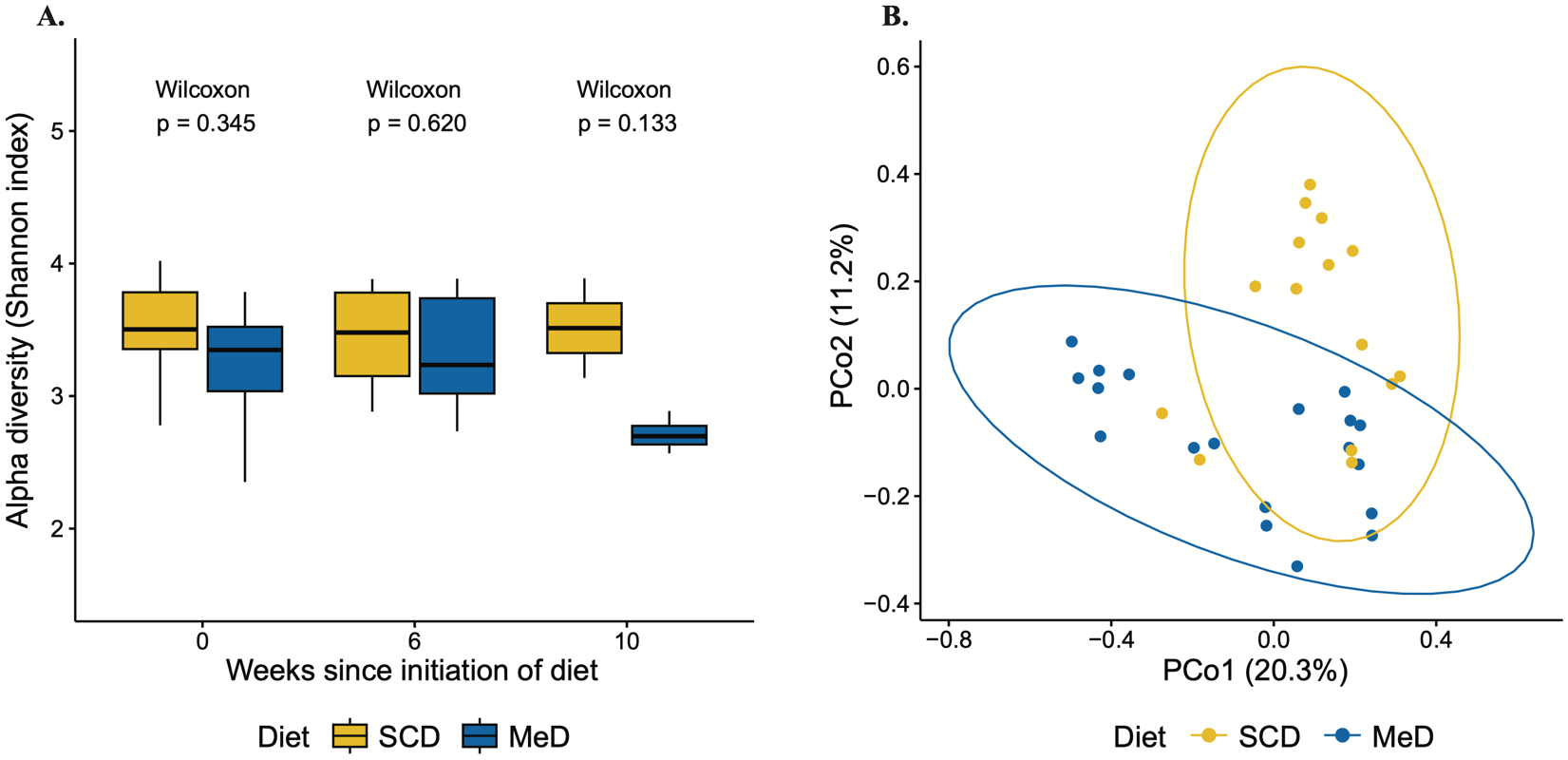


**Figure S3: Proportion of variation explained by individual factors on overall gut microbial community structure**

The numbers above each bar represent the percentage of variation explained by the respective factor. ** P-value < 0.01; * 0.01 ≤ P-value < 0.05.

**Reference:**

1. Lewis JD, Chuai S, Nessel L, Lichtenstein GR, Aberra FN, Ellenberg JH. Use of the noninvasive components of the Mayo score to assess clinical response in ulcerative colitis. Inflamm Bowel Dis. 2008 Dec;14(12):1660-6. eng. The authors report no potential conflicts of interest related to this study. doi:10.1002/ibd.20520. Cited in: Pubmed; PMID 18623174.

2. Irvine EJ, Zhou Q, Thompson AK. The Short Inflammatory Bowel Disease Questionnaire: a quality of life instrument for community physicians managing inflammatory bowel disease. CCRPT Investigators. Canadian Crohn's Relapse Prevention Trial. Am J Gastroenterol. 1996 Aug;91(8):1571-8. eng. Cited in: Pubmed; PMID 8759664.

3. Ware JK, M; Turner-Bowker, M; Gandek, B. How to Score Version 2 of the SF-12 Health Survey. Quality Metric Incorporated, Lincoln; 2002.

4. Ware JH. Interpreting Incomplete Data in Studies of Diet and Weight Loss. New England Journal of Medicine. 2003;348(21):2136-2137. doi:doi:10.1056/NEJMe030054.

5. Kaiser KA, Affuso O, Beasley TM, Allison DB. Getting carried away: a note showing baseline observation carried forward (BOCF) results can be calculated from published complete-cases results. International Journal of Obesity. 2012 2012/06/01;36(6):886-889. doi:10.1038/ijo.2011.25.

6. Proctor LM, Creasy HH, Fettweis JM, Lloyd-Price J, Mahurkar A, Zhou W, Buck GA, Snyder MP, Strauss JF, Weinstock GM, White O, Huttenhower C, The Integrative HMPRNC. The Integrative Human Microbiome Project. Nature. 2019 2019/05/01;569(7758):641-648. doi:10.1038/s41586-019-1238-8.

7. McIver LJ, Abu-Ali G, Franzosa EA, Schwager R, Morgan XC, Waldron L, Segata N, Huttenhower C. bioBakery: a meta'omic analysis environment. Bioinformatics. 2018 Apr 1;34(7):1235-1237. eng. doi:10.1093/bioinformatics/btx754. Cited in: Pubmed; PMID 29194469.

8. Blanco-Míguez A, Beghini F, Cumbo F, McIver LJ, Thompson KN, Zolfo M, Manghi P, Dubois L, Huang KD, Thomas AM, Nickols WA, Piccinno G, Piperni E, Punčochář M, Valles-Colomer M, Tett A, Giordano F, Davies R, Wolf J, Berry SE, Spector TD, Franzosa EA, Pasolli E, Asnicar F, Huttenhower C, Segata N. Extending and improving metagenomic taxonomic profiling with uncharacterized species using MetaPhlAn 4. Nat Biotechnol. 2023 Nov;41(11):1633-1644. eng. S.E.B., T.D.S., F.A. and N.S. are consultants to Zoe Global. F.G, R.D. and J.W. are employees of Zoe Global. The other authors declare no competing interests. Epub 20230223. doi:10.1038/s41587-023-01688-w. Cited in: Pubmed; PMID 36823356.

9. Beghini F, McIver LJ, Blanco-Míguez A, Dubois L, Asnicar F, Maharjan S, Mailyan A, Manghi P, Scholz M, Thomas AM, Valles-Colomer M, Weingart G, Zhang Y, Zolfo M, Huttenhower C, Franzosa EA, Segata N. Integrating taxonomic, functional, and strain-level profiling of diverse microbial communities with bioBakery 3. Elife. 2021 May 4;10. eng. FB, LM, AB, LD, FA, SM, AM, PM, MS, AT, MV, GW, YZ, MZ, CH, EF, NS No competing interests declared. Epub 20210504. doi:10.7554/eLife.65088. Cited in: Pubmed; PMID 33944776.

10. Franzosa EA, McIver LJ, Rahnavard G, Thompson LR, Schirmer M, Weingart G, Lipson KS, Knight R, Caporaso JG, Segata N, Huttenhower C. Species-level functional profiling of metagenomes and metatranscriptomes. Nat Methods. 2018 Nov;15(11):962-968. eng. COMPETING FINANCIAL INTERESTS None declared. Epub 20181030. doi:10.1038/s41592-018-0176-y. Cited in: Pubmed; PMID 30377376.

11. Suzek BE, Huang H, McGarvey P, Mazumder R, Wu CH. UniRef: comprehensive and non-redundant UniProt reference clusters. Bioinformatics. 2007 May 15;23(10):1282-8. eng. Epub 20070322. doi:10.1093/bioinformatics/btm098. Cited in: Pubmed; PMID 17379688.

12. Hua X, McGoldrick J, Nakrour N, Staller K, Chung DC, Xavier RJ, Khalili H. Gut microbiome structure and function in asymptomatic diverticulosis. Genome Med. 2024 Aug 23;16(1):105. eng. HK has received grant funding from Pfizer and Takeda Pharmaceuticals and consulting fees from Abbvie, Takeda, and Aditium Bio. HK also serves on the clinical advisory board of CylindarHealth. KS has received research funding from Ironwood and Urovant and has served as a consultant to Anji, Ardelyx, Arena, Gelesis, Restalsis, Sanofi, and Takeda. RJX is a cofounder of Jnana Therapeutics, Celsius Therapeutics, director of Moonlake Immuno Therapeutics, SAB member at Nestle. None of the industry funding is related to the current study. The remaining authors declare that they do not have any competing interests. Epub 20240823. doi:10.1186/s13073-024-01374-9. Cited in: Pubmed; PMID 39180058.

13. Cohen J. Statistical Power Analysis for the Behavioral Sciences (2nd ed.). Routledge; 1988.

14. Bray JR, Curtis JT. An Ordination of the Upland Forest Communities of Southern Wisconsin. Ecological Monographs. 1957;27(4):325-349. doi:<https://doi.org/10.2307/1942268>.

15. Oksanen J. BFG, Kindt R., Legendre P., Minchin P. R., O’Hara R. B. vegan: Community Ecology Package. R package version 2.6-4. 2022.

16. Mallick H, Rahnavard A, McIver LJ, Ma S, Zhang Y, Nguyen LH, Tickle TL, Weingart G, Ren B, Schwager EH, Chatterjee S, Thompson KN, Wilkinson JE, Subramanian A, Lu Y, Waldron L, Paulson JN, Franzosa EA, Bravo HC, Huttenhower C. Multivariable association discovery in population-scale meta-omics studies. PLoS Comput Biol. 2021 Nov;17(11):e1009442. eng. I have read the journal’s policy and the authors of this manuscript have the following competing interests: CH is on the Scientific Advisory Board for Seres Therapeutics and Empress Therapeutics. The remaining authors have declared that no competing interests exist. Author Yiren Lu was unable to confirm their authorship contributions. On their behalf, the corresponding author has reported their contributions to the best of their knowledge. Epub 20211116. doi:10.1371/journal.pcbi.1009442. Cited in: Pubmed; PMID 34784344.

17. Lloyd-Price J, Arze C, Ananthakrishnan AN, Schirmer M, Avila-Pacheco J, Poon TW, Andrews E, Ajami NJ, Bonham KS, Brislawn CJ, Casero D, Courtney H, Gonzalez A, Graeber TG, Hall AB, Lake K, Landers CJ, Mallick H, Plichta DR, Prasad M, Rahnavard G, Sauk J, Shungin D, Vázquez-Baeza Y, White RA, Bishai J, Bullock K, Deik A, Dennis C, Kaplan JL, Khalili H, McIver LJ, Moran CJ, Nguyen L, Pierce KA, Schwager R, Sirota-Madi A, Stevens BW, Tan W, ten Hoeve JJ, Weingart G, Wilson RG, Yajnik V, Braun J, Denson LA, Jansson JK, Knight R, Kugathasan S, McGovern DPB, Petrosino JF, Stappenbeck TS, Winter HS, Clish CB, Franzosa EA, Vlamakis H, Xavier RJ, Huttenhower C, Investigators I. Multi-omics of the gut microbial ecosystem in inflammatory bowel diseases. Nature. 2019 2019/05/01;569(7758):655-662. doi:10.1038/s41586-019-1237-9.
